# Association of multiple genetic variants with breast cancer susceptibility in the Han Chinese population

**DOI:** 10.18632/oncotarget.13402

**Published:** 2016-11-16

**Authors:** Xu Li, Wenjing Zou, Ming Liu, Wei Cao, Yonghong Jiang, Gaili An, Yuzheng Wang, Shangke Huang, Xinhan Zhao

**Affiliations:** ^1^ Department of Oncology, First Affiliated Hospital, Xi’an Jiatong University, Xi’an, Shannxi 710061, China; ^2^ Department of The First of Internal Medicine, Tumor Hospital of Shaanxi Province Affiliated Hospital, Medical College of Xi’an Jiao Tong University, Xi’an, Shannxi 710061, China; ^3^ Department of The Fifth of Internal Medicine, Xi’an No5 Hospital, Xi’an, Shannxi 710082, China; ^4^ Department of Obstetrics and Gynecology, Second Affiliated Hospital, Xi’an Jiatong University, Xi’an, Shannxi 710003, China; ^5^ Department of Oncological Surgery, Shaanxi Provincial People's Hospital, Xi’an 710068, China; ^6^ Department of Oncological Surgery, Weinan Central Hospital, Weinan, Shaanxi 714000, China; ^7^ Department of Oncology, Shaanxi Provincial People's Hospital, Xi’an 710068, China

**Keywords:** breast cancer, single nucleotide polymorphism (SNP), case-control study, Chinese population

## Abstract

We selected 13 tag single nucleotide polymorphisms (tSNPs) to investigate whether they were associated with breast cancer risk in the Chinese Han population. Upon statistical analyses of clinical data from 551 patients and 577 controls, we found that six of the 13 SNPs were associated with breast cancer; namely, rs4973768(Odds ratio (OR) = 1.30, 95% confidence interval (CI) =1.01-1.67), rs981782(OR =1.30, 95% CI=1.01-1.66), rs1432679(OR =0.84, 95% CI=0.70-0.99), rs10759243(OR=1.30, 95%CI=1.09-1.55), rs10822013(OR =1.18, 95% CI=1.00-1.39) and rs704010(OR =1.63, 95% CI=1.04-2.56). When stratified based on breast cancer subtype, our analyses revealed that three SNPs (rs981782, rs10759243 and rs704010) correlated with ER+ breast cancer, while another three (rs4973768, rs1432679 and rs10822013) correlated with ER- breast cancer. We obtained similar results while investigating the correlation of SNPs with PR status or clinical stage. Our results suggest that associations identified between SNPs and breast cancer through genome-wide association studies (GWAS) may not always be generalizable across races.

## INTRODUCTION

Of all the cancers types, breast cancer is leading to the highest incidence and mortality rate in Europe [1] and accounts for ~23% of new cancer cases as well as ~14% of cancer-related death [2]. The cause of etiology it is not clear, studying suggested that breast cancer(BC) is a complex disease. Recently, single nucleotide polymorphisms (SNP) in some key loci genetic variations can lead to differences in BC susceptibility between persons. But, most genome-wide association studies (GWAS) focused on women of European ancestry. Since the minor allele frequencies and the linkage disequilibrium patterns of the SNP loci were significantly different in different ethnicities, performing GWAS in populations of different racial descent could help to determine whether previous conclusions regarding the association between SNPs and breast cancer susceptibility are generalizable. Recently, for take disease heterogeneity into account following GWAS analyses, the GWAS-MPE (molecular pathological epidemiology) method was raised [3, 4]. This approach may provide causal link between risk variants and molecular signatures in diseased cells, thereby allowing to estimate risk for each molecular subtype more precisely and to identify new variant-subtype relationships.

BC is a heterogeneous cancer in which different risk factors may be leading to different tumor subtypes and that performanced different biological behaviors and progression. Indeed, BC hormone receptor status and pathologic features of cancer affect risk factors. For example, common genetic variants with estrogen receptor (ER + and ER -) breast cancer differences related to support ER + and ER - diseases caused by different etiology way hypothesis [5-7]. Estrogen receptor (ER) and progesterone receptor (PR) are the most commonly used biomarkers for breast cancer subtyping. However, different intrinsic subtypes have significantly in incidence, survival and response to therapy [8, 9]. Since different subtypes of the tumor, the risk of breast cancer is not the same, here we evaluated breast cancer patients in China for 13 SNPs recently identified from GWAS. Namely, we evaluationed the associations of the SNPs with BC ER status, PR status and Clinic stage.

## RESULTS

The 551 BC cancers and 577 normal personss the basic information were listed in Table 1, in Table S1 we listed the MAF of all SNP, stratified by ER and PR status and Table S3 we listed the MAF of all SNP, stratified by Clinic stage. However, the cases BMI were significantly lower than the normal groups (*p* =0.027). We carried out the correction for age and BMI in the follow-up multivariate logistic regression analyses. The study found that one locus (rs1432679, *p*=0.043) in the *EBF1* gene, another one (rs10759243, *p* =0.012) in the *KLF4-RPL36AP6* gene, and yet another (rs10822013, *p* =0.046) in *ZNF365* gene were association with breast cancer based on χ2 tests (Table 2).

**Table 1:** Clinical characteristics and Disease characteristics of the case–control study population

Variables	n	Case (%)	n	Control (%)	P
Age	551	49.09±11.022	577	48.79±8.294	0.613^a^
BMI	459	22.52±2.837	549	22.95±3.214	0.027^b^
Estrogen receptor (ER) status					
Positive	292	53			
Negative	136	24.7			
Unknown	123	22.3			
Progesterone receptor (PR) status					
Positive	247	44.8			
Negative	180	32.7			
Unknown	124	22.5			
Clinic stage(UICC)					
1-2	348	63.2			
3-4	148	26.9			
Unknown	55	10			

a P value was calculated by Welch's t test, p<0.05 indicates statistical significance.

b P value was calculated by Student's t test, p<0.05 indicates statistical significance.

**Table 2:** Allele frequencies in cases and controls and odds ratio estimates for breast cancer

SNP	Gene (s)	position	Alleles A/B	MAF		p-HWE			*P*
				Case	Control		Rs	95%CI	
rs4849887	LINC01101-GLI2	121245122	T/C	0.200	0.800	0.889	1.138	0.92-1.40	0.225
rs6762644	ITPR1	4742276	G/A	0.090	0.910	0.805	0.991	0.75-1.32	0.951
rs4973768	SLC4A7	27416013	T/C	0.250	0.750	0.906	1.131	0.93-1.37	0.212
rs981782	HCN1	45285718	G/T	0.354	0.646	0.341	1.163	0.98-1.38	0.088
rs16886165	RPL26P19-MAP3K1	56023083	G/T	0.312	0.688	0.925	0.940	0.79-1.12	0.489
rs889312	RPL26P19-MAP3K1	56031884	A/C	0.504	0.496	0.741	1.025	0.87-1.21	0.769
rs1432679	EBF1	158244083	T/C	0.325	0.675	0.929	0.836	0.70-0.99	0.043*
rs2180341	RNF146	127600630	G/A	0.260	0.740	0.514	1.007	0.83-1.22	0.944
rs10759243	KLF4 - RPL36AP6	110306115	A/C	0.476	0.524	0.270	1.238	1.05-1.46	0.012*
rs10822013	ZNF365	64251977	T/C	0.488	0.512	0.933	1.182	1.00-1.39	0.046*
rs704010	ZMIZ1	80841148	A/G	0.320	0.680	0.052	1.185	0.99-1.42	0.062
rs10771399	PTHLH - CCDC91	28155080	G/A	0.181	0.819	0.884	1.063	0.86-1.32	0.579
rs17356907	USP44-PGAM1P5	96027759	G/A	0.230	0.770	0.820	0.947	0.78-1.15	0.582

A/B: Minor/major alleles on the control sample; *p value ≤ 0.05 indicates statistical significance; HWE, Hardy-Weinberg Equilibrium; ORs, odds ratios.

We also performed other genetic models analyzed on the relationship between SNPs and BC risk (Table 3). We observed that the minor alleles of rs10759243, rs4973768, rs981782 and rs704010 were associated with breast cancer risk. In the codominant model of rs10759243, the genotype “C/A” and “A/A” increased BC risk by 1.45-fold (OR = 1.45; 95% CI, 1.08-1.94; *p* = 0.01), 1.65-fold (OR = 1.65; 95% CI, 1.15-2.37; *p* = 0.01), respectively, “C/A-A/A” genotype in the dominant model (OR, 1.50; 95 % CI, 1.14-1.98; *p* = 0.0035). Additionally, the genotypes “C/T-T/T” of rs4973768 (OR = 1.30; 95% CI, 1.01-1.67; *p* = 0.041) and “G/T-G/G” of rs981782 indicated an increased the BC risk in the dominant model (OR = 1.30, 95% CI, 1.01 – 1.66, *p* = 0.043). Meanwhile, the genotype “A/A” of rs704010 may increase breast cancer risk in the recessive model (OR = 1.63, 95% CI, 1.04 - 2.56, *p* = 0.033).

**Table 3:** Logistic regression analysis of the association between the SNPs and breast cancer risk

SNP	Model	Genotype	Control	Case	OR(95%CI)	P-value	AIC	BIC
rs4973768		C/C	329(59.9%)	246(53.6%)	1.00			
	Codominant	C/T	194(35.3%)	190(41.4%)	1.31(1.01-1.70)	0.12	1389.8	1414.4
		T/T	26 (4.7%)	23 (5%)	1.21(0.67-2.17)			
	Dominant	C/C	329(59.9%)	246(53.6%)	1.00			
		C/T-T/T	220(40.1%)	213(46.4%)	1.30(1.01-1.67)	0.041	1387.9	1407.6
	Recessive	C/C-C/T	523(95.3%)	436 (95%)	1.00			
		T/T	26 (4.7%)	23 (5%)	1.08(0.61-1.93)	0.79	1392	1411.7
	Log-additive	---	---	---	1.22(0.98-1.50)	0.069	1388.8	1408.5
rs10759243		C/C	190(34.7%)	119(26.6%)	1.00			
	Codominant	C/A	257(46.9%)	228(50.9%)	1.45 (1.08-1.94)	0.01	1365.6	1390.1
		A/A	101(18.4%)	101(22.5%)	1.65 (1.15-2.37)			
	Dominant	C/C	190(34.7%)	119(26.6%)	1.00	0.0035	1364.3	1383.9
		C/A-A/A	358(65.3%)	329(73.4%)	1.50 (1.14-1.98)			
	Recessive	C/C-C/A	447(81.6%)	347(77.5%)	1.00	0.083	1369.8	1389.4
		A/A	101(18.4%)	101(22.5%)	1.32 (0.96-1.80)			
	Log-additive	---	---	---	1.30(1.09-1.55)	0.0038	1364.4	1384.1
rs981782		T/T	263(47.9%)	189(41.2%)	1.00			
	Codominant	G/T	226(41.2%)	223(48.6%)	1.35(1.04-1.76)	0.078	1389	1413.6
		G/G	60 (10.9%)	47 (10.2%)	1.08 (0.71-1.66)			
	Dominant	T/T	263(47.9%)	189(41.2%)	1.00			
		G/T-G/G	286(52.1%)	270(58.8%)	1.30(1.01-1.66)	0.043	1388	1407.7
	Recessive	T/T-G/T	489(89.1%)	412(89.8%)	1.00			
		G/G	60 (10.9%)	47 (10.2%)	0.93 (0.62-1.40)	0.74	1392	1411.6
	Log-additive	---	---	---	1.14 (0.94-1.38)	0.17	1390.2	1409.9
rs704010		G/G	273(49.7%)	212(46.2%)	1.00			
	Codominant	G/A	240(43.7%)	199(43.4%)	1.08 (0.83-1.40)	0.088	1389.2	1413.8
		A/A	36 (6.6%)	48 (10.5%)	1.69(1.06-2.70)			
	Dominant	G/G	273(49.7%)	212(46.2%)	1.00			
		G/A-A/A	276(50.3%)	247(53.8%)	1.16 (0.91-1.49)	0.24	1390.7	1410.4
	Recessive	G/G-G/A	513(93.4%)	411(89.5%)	1.00			
		A/A	36 (6.6%)	48 (10.5%)	1.63(1.04-2.56)	0.033	1387.6	1407.2
	Log-additive	---	---	---	1.20 (0.99-1.46)	0.065	1388.7	1408.4

*P*: adjusted by Age and BMI; AIC: Akaike's Information criterion; BIC: Bayesian Information criterion.

*p value ≤ 0.05 indicates statistical significance.

### Association between SNPs and ER status

SNPs rs10759243 in the *KLF4* gene (OR = 1.333, 95% CI, 1.090 – 1.629, *p* = 0.005), rs704010 in the *ZMIZ1* gene (OR = 1.304, 95% CI, 1.053 – 1.614, *p* = 0.015) are associated with an increased the ER+ BC risk (Supplementary Table S2). We also found an increased risk of ER+ breast cancer For SNP rs10759243 (*P*<0.003 for all analyses except the recessive model) with the highest OR equal to 1.88 (1.24–2.86) for the “A/A” genotype in the codominant model (Supplementary Table S5). Similarly, SNP rs704010 was observed (*P*<0.002 for all analyses except the dominant model) with the highest OR equal to 2.08 (1.25–3.45) for the “A/A” genotype in the codominant model. Although no significant association was observed between rs981782 in the *NCN1* gene and ER+ breast cancer risk in allelic model analysis, this SNP showed a strong association with ER positive cancer in genetic models.

The SNPs rs4973768 in the *SLC4A7* gene (OR = 1.442, 95% CI, 1.075 – 1.934, *p* = 0.014) and rs10822013 in the *ZNF365* gene (OR = 1.313, 95% CI, 1.008 –1.710, *p* = 0.043) were associated with the risk of ER- BC (Supplementary Table S2). In genetic model analyses, rs4973768 may increase ER- breast cancer risk (*p* =0.022) in the dominant model of the “C/T-T/T” genotype and (*p* =0.016) in the additive model (Supplementary Table S6). Furthermore, rs10822013 may increase ER- breast cancer risk (*p* =0.035) in the additive model. However, The SNP rs1432679 in the *EBF1* gene reduced the ER- BC risk(OR = 0.737, 95% CI, 0.553 –0.980, *p* = 0.036) and in the log-additive model (*p* = 0.043).

### Association between SNPs and PR status

We identified an increased the risk of PR+ BC for SNP rs10759243 (*P*<0.002 for all analyses except the recessive model) with the highest OR equal to 1.65 (1.05–2.58) for the “A/A” genotype in the codominant model (Supplementary Table S2). The SNP rs704010 in the *ZMIZ1* gene was associated with an increased the risk of PR+ BC (OR = 1.288, 95% CI, 1.028 – 1.615, *p* = 0.028), in the dominant model (OR of 1.38 (1.02–1.87), *p* = 0.037) for the genotype G/A-A/A and in the log-additive model (OR of 1.32(1.04–1.67), *p* = 0.024) (Supplementary Table S7). Although we found no association between the rs981782 SNP in the *NCN1* gene and PR+ BC risk in allelic model analysis, the results indicated that this SNP was associated with PR positive cancer (*p* = 0.02 for the dominant model, with an OR of 1.44) for the genotype G/T-G/G.

The SNP rs1432679 in the *EBF1* gene reduced the risk of \ PR- BC risk (OR = 0.715, 95% CI, 0.553 - 0.925, *p* = 0.010) and in the codominant model (OR of 0.52 (0.28–0.96), *p* = 0.047) for the genotype T/T (Supplementary Table S8), as well as in the dominant model (OR of 0.68 (0.48–0.95), *p* = 0.026 ) for the genotype T/C -T/T and the log-additive model (OR of 0.72 (0.56–0.94), *p* = 0.014). The SNP rs10759243 in the *KLF4* gene was associated with the PR- BC risk (OR = 1.315, 95% CI, 1.036–1.671, *p* = 0.024) and in the log-additive model (OR = 1.33, 95% CI, 1.04 – 1.68, *p* = 0.021).

### Association between SNPs and clinical stage

The SNP rs1432679 in the *EBF1* gene provided a protective effect in terms of clinical stage (UICC) I and II BC (OR = 0.803, 95% CI, 0.658 - 0.980, *p* = 0.031) (Supplementary Table S4). On the other hand, genetic models showed no association with clinical stage (UICC) I and II cancer. The SNPs rs10759243 in the *KLF4* gene (OR = 1.284, 95% CI, 1.061 – 1.552, *p* = 0.010) and rs704010 in the *ZMIZ1* gene (OR = 1.237, 95% CI, 1.010 – 1.515, *p* = 0.040) were associated with the risk of clinical stage (UICC) I and II breast cancer compared with normal controls (Supplementary Table S9). Such increased risk for the variant rs10759243 in the *KLF4* gene was confirmed in all the analyses, with the highest OR equal to 1.74 (1.17-2.61) for the “A/A” genotype in the codominant model. For SNP rs704010, the genotype “A/A” increased clinical stage (UICC) I and II breast cancer risk by 1.90-fold (OR = 1.90; 95% CI, 1.15-3.15; *p* = 0.042) in the codominant model and1.88-fold (OR = 1.88; 95% CI, 1.15-3.05; *p* = 0.031) in the recessive model.

For SNP rs981782 in the *HCN1* gene, the genotype “G/T” increased clinical stage (UICC) III and IV breast cancer risk by1.83-fold (OR = 1.83; 95% CI, 1.20-2.80; *p* = 0.063) in the codominant model and “G/T-G/G” genotype in the dominant model (OR = 1.62; 95% CI, 1.08-2.45; *p* = 0.019) (Supplementary Table S4).

For SNP rs10759243 in the *KLF4* gene, the genotype “C/A” increased clinic stage (UICC) III and IV breast cancer risk by1.85-fold (OR = 1.85; 95% CI, 1.14-3.00; *p* = 0.037) in the codominant model and “C/A-A/A” genotype in the dominant model (OR = 1.79; 95% CI, 1.12-2.85; *p* = 0.012) (Supplementary Table S10). Additionally, a marginal association (*p* = 0.05) was found between the variant rs4849887 in the *LINC01101-GLI2* gene and an increased risk for clinic stage (UICC) III and IV breast cancer in the recessive model, with an OR of 2.39 (1.04-5.46) for the genotype T/T.

## DISCUSSION

We genotyped 13 tSNPs previously reported to be associated with breast cancer. Our analyses revealedthere have seven SNPswere associated the BC risk. These tSNPs correlated to different degrees with ER/PR status and clinical stage. Our results suggested that these gene polymorphisms may be associated with the risk of BC in Chinese women.

The Kruppel-like factor (KLF) family is a group of transcription factors. And they can control cells of various physiological activities, such as proliferation, apoptosis, inflammation and so on. In particular, the KLF4 gene contributes to maintaining CSCs and promotes migration and invasion, resulting in tumor formation *in vivo* [10]. KLF4 has been reported to act as both an oncogene and a tumor suppressor in breast cancer [11]. KLF4 specific mechanism we are not yet clear. The SNP *rs10759243* in the *KLF4* gene was associated with the risk of BC in European and Chinese populations [12]. Our study confirmed that SNP rs10759243 was association with BC in Chinese, in agreement with previous findings. Furthermore, we found that this SNP is also associated with ER+ and PR+ Breast cancer subtypes, consistent with previous results [12].

The transcription factor EBF1 is an interaction partner of the *TET2* gene and is involved in the regulation of DNA methylation in a tissue- and sequence-specific mode of IDH1-mutant cancers [13]. In addition, many studies [14, 15] have found mammographic density are associated with BC risk. The association of SNP rs1432679 (*EBF1* gene) with breast cancer appears to correlate with mammographic density phenotypes. Namely, Jennifer Stone's study indicated that the minor “G” allele of rs1432679 is positively association with dense breast tissue and negatively association with non-dense tissue, and was positively associated with percent density (*p*=1.1x10-5). Furthermore, this SNP correlated with increased the BC risk in Europeans (OR = 1.06; *p* = 0.002) [7]. On the other hand, Zhang Bo's study of the Chinese population showed that rs1432679 provided a protective effect against breast cancer (OR= 0.98) [12]. Our results are consistent with Zhang Bo's research (OR = 0.836; *p* = 0.043). In addition, our research here also found that SNP rs1432679 provided a protective effect against ER- and PR- breast cancer.

The SNP rs704010, lies in the intron 1 of the *ZMIZ1* gene, it is encoding zinc finger MIZ-type containing 1, and regulated the activity of various transcription factors [16]. The androgen receptor (AR) is widespread sex steroid receptor in *in situ*, invasive, and metastatic BC [17]. Smads are identified proteins that mediate intracellular signaling of TGF-β1. TGFβ1-activated TBX3 protein expression is mediated by Smad3/4 and TBX3 is overexpressed in several cancers, including breast cancer [18]. P53 protein is involved in various cellular processes, such as cell senescence, expression and so on [19, 20]. The p53 gene expression of correlates with BC prognosis [21, 22]. Previously, the SNP *rs704010* in the *ZMIZ1* gene was found to correlate with an increased risk of breast cancer (OR = 1.03) and ER+ BC (OR = 1.02) in women with European ancestry [23]. Turnbull *et al.* also found rs704010 that the minor allele of “A“have significantly association with BC risk (per-allele OR = 1.38 for stage 1; per-allele OR = 1.13 for stage 2; Combined *p* = 3.7*10-9) [24].

The *SNP rs2180341* in the *RNF146* gene at 6q21.33 also correlates with the BC risk in Europeans [25, 26]. However, no significant association was observed in several studies involving Asians [27-30], with the exception of the study by Jun Cheng Dai *et al.* [31]. Our results for Chinese women were consistent with those from the majority of reports focusing on Asian populations.

The *Rs889312* SNP in the *RPL26P19-MAP3K1* gene was successfully replicated in Korean women with breast cancer [32]. However, it was not correlates with BC in Chinese women [28, 30, 31, 33, 34], in agreement with our results here.

The rs10822013 SNP at 10q21.2 is located in an intronic region of the zinc finger protein 365 (ZNF365). Qiuyin Cai *et al.* [35] found among East-Asian women that the SNP is a risk variant for BC and the OR = 1.10 (95% CI: 1.07-1.14) (P = 5.87 * 10^-9^). Qin Z *et al.* [36] found that rs10771399 were also associated with BC risk in Asian women. Michcailidou K *et al.* recently found the SNP rs4849887 (2q14/INHBB) [37], and which was not evaluated in previous studies of African-American women. The rs16886165 SNP, which is close to 3’ of the MAP3K1 gene, was foundassociation with breast cancer risk in White women [38].

The other five SNPs we analyzed (*rs4849887/LINC01101-GLI2* gene, *rs6762644/ITPR1* gene, *rs16886165/RPL26P19-MAP3K1* gene, *rs10771399/PTHLH - CCDC91* gene, *rs17356907/USP44-PGAM1P5* gene) were not correlates with BC risk in our research. Potential explanations for the difference when compared to previous results could be the effects of other risk factors, or interactions between genes, interactions between genes and the environment, and the effects of LD and the highly variable minor allele frequencies of SNPs in different ethnicities. Our study suffered from other limitations. For example, we only evaluated a limited number of breast cancer associated with risk factors, which did not include age at menarche, age at first live birth and family history of BC. Furthermore, because of our relatively small sample size, body mass index (BMI) was not matched across cases and controls. However, we successfully replicated 13 SNPs from GWAS identified were asscciated with BC in Chinese people breast cancer cases and matched controls. Our study highlight the need to assess the generalizability of previously identified associations between SNPs and BC and verification is required in different populations.

## MATERIALS AND METHODS

### Study population

A case-control study was conducted to assess genetic associations with breast cancer risk. Eligible cases were those newly diagnosed with breast cancer at the Second Affiliated Hospital, Xi’an Jiatong University, and the Shaanxi Provincial People's Hospital, Xi’an City, China, between June 2012 and Dec 2014. The controls were cancer-free patients randomly recruited from the health centers of the two hospitals during the same study period, and were matched with cases based on age and ethnicity. All cases had histologically confirmed breast cancer. We excluded cases that underwent radiotherapy or chemotherapy as well as controls with chronic disease. All subjects were at least 18 years old and were in good mental condition. Additionally, all of the cases and the controls were genetically unrelated, ethnic Han Chinese women. In total, 577 controls (all female; median age 48.79±8.294 years) and 551 breast cancer cases (all female; median age 49.09±11.022 years) were recruited in this study.

### Clinical data and demographic information

Each participant filled in a standard questionnaire to record demographic information including age, BMI, estrogen receptor (ER), progesterone receptor (PR), clinical stage and family history of cancer. All subjects signed informed consent forms. Blood (5 ml) was collected from each subject according to the study protocol approved by the Clinical Research Ethics Committee of the Second Affiliated Hospital, Xi’an Jiatong University, and the Clinical Research Ethics Committee of the Shaanxi Provincial People's Hospital.

### tSNP selection and genotyping

A SNPs were selected from based on a review of published literature to be associated with Breast Cancer [7, 12, 35, 36, 39-43] and with minor allele frequencies (MAF) of >5% in the HapMap of the Chinese Han Beijing (CHB) population were selected based on a review of published literature. Genomic DNA was isolated from whole blood samples using the GoldMag-Mini Purification Kit (GoldMag Co. Ltd. Xian city, China). DNA concentrations were measured using the NanoDrop2000 (Thermo Scientific, Waltham, Massachusetts, USA). Sequenom MassARRAY Assay Design 3.0 software was used to design a multiplexed SNP MassEXTENDED assay. A Sequenom MassARRAY mass spectrometry analyzer (Sequenom, San Diego, CA, USA) was used for genotyping and data were managed using Sequenom Typer 4.0 Software (Sequenom, San Diego, CA, USA) [44, 45].

### Statistical analyses

Hardy-Weinberg equilibrium (HWE) was assessed in the control samples by applying an exact test. Deviation from HWE was considered significant at the *P*<0.05 level. Next, the allelic frequencies of each tSNP were compared in cases versus controls using the x2 test [46]. Finally, the association between each tSNP and breast cancer was assessed under multiple inheritance models using SNPStats, a web-based program available at http://bioinfo.iconcologia.net/snpstats/start.htm [47]. Unconditional logistic regression model adjusted for age and BMI was used to calculate odds ratios (ORs) and 95% confidence intervals (CI) for each polymorphism. Akaike's information criteria (AIC) and the Bayesian information criteria (BIC) were used to determine the best-fit model for each tSNP. We also analyzed the association between each tSNP and breast cancer after stratification by ER status, PR status and clinical stage to assess whether there was a significant difference in the probability of developing breast cancer under different disease conditions, using the SNP stats software. All statistical tests were two-sided, and a *P* value equal to or less than 0.05 was considered significant.

## SUPPLEMENTARY MATERIALS FIGURES AND TABLES




