# Supplementary material for: Association of multiple genetic variants with breast cancer susceptibility in the Han Chinese population
Source: Oncotarget. 2016 Nov 16;7(51):85483–91. doi: 10.18632/oncotarget.13402 (PMC5356751; doi:10.18632/oncotarget.13402)
Supplement: Supplementary file 1 [file oncotarget-07-85483-s001.pdf]

Association of multiple genetic variants with breast cancer susceptibility in the Han Chinese population

SUPPLEMENTARY TABLES

Supplementary Table S1: The MAF of all SNP, stratified by ER and PR status

SNP	Allele	ER+ (MAF)		ER- (MAF)		PR+(MAF)		PR-(MAF)	
	A/B	Case	Control	Case	Control	Case	Control	Case	Control
rs4849887	T/C	0.199	0.180	0.184	0.180	0.194	0.180	0.194	0.180
rs6762644	G/A	0.096	0.091	0.077	0.091	0.085	0.091	0.097	0.091
rs4973768	T/C	0.233	0.227	0.298	0.227	0.239	0.227	0.272	0.227
rs981782	G/T	0.360	0.320	0.309	0.320	0.360	0.320	0.317	0.320
rs16886165	G/T	0.331	0.326	0.316	0.326	0.321	0.326	0.335	0.326
rs889312	A/C	0.490	0.497	0.504	0.497	0.500	0.497	0.483	0.497
rs1432679	T/C	0.336	0.365	0.298	0.365	0.348	0.365	0.292	0.365
rs2180341	G/A	0.267	0.259	0.233	0.259	0.255	0.259	0.491	0.424
rs10759243	A/C	0.495	0.424	0.462	0.424	0.479	0.424	0.314	0.284
rs10822013	T/C	0.454	0.447	0.515	0.447	0.460	0.447	0.178	0.172
rs704010	A/G	0.341	0.284	0.301	0.284	0.338	0.284	0.269	0.240
rs10771399	G/A	0.180	0.172	0.169	0.172	0.174	0.172	0.194	0.180
rs17356907	G/A	0.219	0.240	0.254	0.240	0.200	0.240	0.097	0.091

A/B: Minor/major alleles on the control sample; MAF:Minor Allele Frequency.

Supplementary Table S2: Associations of All SNP with breast cancer risk, stratified by ER and PR status (adjusted by Age+BMI)

See Supplementary File 1

Supplementary Table S3: The MAF of all SNP, stratified by Clinic stage

SNP	Alleles	I and II combined (MAF)		III and IV combined (MAF)	
	A/B	Case	Control	Case	Control
rs4849887	T/C	0.195	0.180	0.196	0.180
rs6762644	G/A	0.088	0.091	0.105	0.091
rs4973768	T/C	0.248	0.227	0.245	0.227
rs981782	G/T	0.346	0.320	0.365	0.320
rs16886165	G/T	0.306	0.326	0.324	0.326
rs889312	A/C	0.510	0.497	0.493	0.497
rs1432679	T/C	0.316	0.365	0.348	0.365
rs2180341	G/A	0.260	0.259	0.253	0.259
rs10759243	A/C	0.485	0.424	0.473	0.424
rs10822013	T/C	0.477	0.447	0.486	0.447
rs704010	A/G	0.329	0.284	0.318	0.284
rs10771399	G/A	0.181	0.172	0.179	0.172
rs17356907	G/A	0.224	0.240	0.233	0.240

A/B: Minor/major alleles on the control sample; MAF:Minor Allele Frequency.

Supplementary Table S4: Associations of All SNP with breast cancer risk, stratified by Clinic stage (adjusted by Age + BMI)

SNP	Alleles	I and II combined		III and IV combined	
	A/B	OR(95% CI)	*p* value	OR(95% CI)	*p* value
rs4849887	T/C	1.106(0.870-1.405)	0.412	1.109(0.803-1.533)	0.529
rs6762644	G/A	0.961(0.691-1.336)	0.811	1.170(0.767-1.784)	0.466
rs4973768	T/C	1.120(0.899-1.396)	0.312	1.103(0.818-1.487)	0.522
rs981782	G/T	1.126(0.923-1.374)	0.242	1.221(0.935-1.595)	0.141
rs16886165	G/T	0.914(0.746-1.119)	0.382	0.993(0.756-1.304)	0.959
rs889312	A/C	1.052(0.872-1.269)	0.598	0.983(0.762-1.269)	0.898
rs1432679	T/C	0.803(0.658-0.980)	0.031*	0.927(0.710-1.211)	0.578
rs2180341	G/A	1.006(0.811-1.248)	0.958	0.971(0.723-1.303)	0.842
rs10759243	A/C	1.284(1.061-1.552)	0.010*	1.220(0.943-1.578)	0.130
rs10822013	T/C	1.129(0.935-1.363)	0.206	1.173(0.908-1.514)	0.221
rs704010	A/G	1.237(1.010-1.515)	0.040*	1.174(0.891-1.547)	0.254
rs10771399	G/A	1.061(0.830-1.357)	0.635	1.047(0.750-1.462)	0.787
rs17356907	G/A	0.914(0.731-1.143)	0.430	0.962(0.712-1.300)	0.800

*p value ≤ 0.05 indicates statistical significance.

Supplementary Table S5: Logistic regression analysis of the association between the SNPs and ER+ breast cancer risk (adjusted by Age + BMI)

See Supplementary File 1

Supplementary Table S6: Logistic regression analysis of the association between the SNPs and ER-breast cancer risk (adjusted by Age + BMI)

See Supplementary File 1

Supplementary Table S7: Logistic regression analysis of the association between the SNPs and PR+ breast cancer risk (adjusted by Age + BMI)

See Supplementary File 1

Supplementary Table S8: Logistic regression analysis of the association between the SNPs and PR- breast cancer risk (adjusted by Age + BMI)

See Supplementary File 1

Supplementary Table S9: Logistic regression analysis of the association between the SNPs and Clinic stage (UICC) I and II breast cancer risk (adjusted by Age + BMI)

See Supplementary File 1

Supplementary Table S10: Logistic regression analysis of the association between the SNPs and Clinic stage (UICC) III and IV breast cancer risk (adjusted by Age + BMI)

See Supplementary File 1
