# Supplementary material for: Association of multiple genetic variants with breast cancer susceptibility in the Han Chinese population
Source: Oncotarget. 2016 Nov 16;7(51):85483–91. doi: 10.18632/oncotarget.13402 (PMC5356751; doi:10.18632/oncotarget.13402)
Supplement: Supplementary file 2 [file oncotarget-07-85483-s002.doc]

**Table S2** Associations of All SNP with breast cancer risk, stratified by ER and PR status (adjusted by Age+BMI).

| SNP | ER+ | | ER- | | PR+ |  | PR- |  |
| --- | --- | --- | --- | --- | --- | --- | --- | --- |
|  | ORs(95% CI) | *p* | ORs(95% CI) | *p* | ORs(95% CI) | *p* | ORs(95%CI) | *p* |
| *p* rs4849887 | 1.128(0.877-1.452) | 0.348 | 1.025(0.729-1.442) | 0.886 | 1.098(0.840-1.436) | 0.494 | 1.099(0.813-1.484) | 0.539 |
| rs6762644 | 1.061(0.755-1.490) | 0.735 | 0.837(0.514-1.363) | 0.473 | 0.929(0.639-1.351) | 0.700 | 1.077(0.721-1.609) | 0.718 |
| rs4973768 | 1.032(0.815-1.307) | 0.793 | 1.442(1.075-1.934) | 0.014* | 1.067(0.833-1.367) | 0.608 | 1.272(0.971-1.665) | 0.080 |
| rs981782 | 1.194(0.969-1.471) | 0.097 | 0.950(0.714-1.263) | 0.724 | 1.198(0.960-1.494) | 0.110 | 0.985(0.764-1.270) | 0.908 |
| rs16886165 | 1.024(0.828-1.256) | 0.830 | 0.956(0.720-1.270) | 0.758 | 0.978(0.781-1.226) | 0.850 | 1.043(0.811-1.341) | 0.743 |
| rs889312 | 0.970(0.795-1.183) | 0.761 | 1.025(0.787-1.335) | 0.853 | 1.010(0.819-1.247) | 0.924 | 0.945(0.746-1.197) | 0.640 |
| rs1432679 | 0.877(0.712-1.082) | 0.220 | 0.737(0.553-0.980) | 0.036* | 0.928(0.745-1.156) | 0.505 | 0.715(0.553-0.925) | 0.010* |
| rs2180341 | 1.043(0.832-1.309) | 0.714 | 0.869(0.635-1.189) | 0.379 | 0.979(0.768-1.249) | 0.866 | 0.989(0.753-1.299) | 0.938 |
| rs10759243 | 1.333(1.090-1.629) | 0.005* | 1.169(0.894-1.529) | 0.253 | 1.253(1.013-1.550) | 0.038* | 1.315(1.036-1.671) | 0.024* |
| rs10822013 | 1.028(0.842-1.256) | 0.783 | 1.313(1.008-1.710) | 0.043* | 1.053(0.852-1.300) | 0.635 | 1.184(0.935-1.500) | 0.161 |
| rs704010 | 1.304(1.053-1.614) | 0.015 | 1.089(0.816-1.453) | 0.564 | 1.288(1.028-1.615) | 0.028* | 1.154(0.893-1.491) | 0.273 |
| rs10771399 | 1.052(0.811-1.365) | 0.700 | 0.977(0.688-1.389) | 0.898 | 1.012(0.767-1.336) | 0.933 | 1.038(0.761-1.415) | 0.813 |
| rs17356907 | 0.888(0.700-1.127) | 0.328 | 1.076(0.793-1.458) | 0.639 | 0.793(0.613-1.027) | 0.078 | 1.167(0.892-1.527) | 0.260 |

**p* value ≤ 0.05 indicates statistical significance,

**Table S5** Logistic regression analysis of the association between the SNPs and ER+ breast cancer risk (adjusted by Age + BMI).

| **SNP** | **Model** | | **Genotype** | | **Control** | | **Case** | | **OR(95%CI)** | | **P-value** | | **AIC** | | **BIC** | |  |
| --- | --- | --- | --- | --- | --- | --- | --- | --- | --- | --- | --- | --- | --- | --- | --- | --- | --- |
| rs981782 |  | | T/T | | 263(47.9%) | | 113(38.7%) | | 1.00 | |  | |  | |  | |  |
|  | Codominant | | G/T | | 226(41.2%) | | 148(50.7%) | | **1.50(1.11-2.04)** | | 0.03* | | 1081.5 | | 1105.2 | |  |
|  |  | | G/G | | 60 (10.9%) | | 31 (10.6%) | | 1.21 (0.74-1.97) | |  | |  | |  | |  |
|  | Dominant | | T/T | | 263(47.9%) | | 113(38.7%) | | 1.00 | |  | |  | |  | |  |
|  |  | | G/T-G/G | | 286(52.1%) | | 179(61.3%) | | **1.44(1.08-1.93)** | | 0.013* | | 1080.3 | | 1099.3 | |  |
|  | Recessive | | T/T-G/T | | 489(89.1%) | | 261(89.4%) | | 1.00 | |  | |  | |  | |  |
|  |  | | G/G | | 60 (10.9%) | | 31 (10.6%) | | 0.98 (0.62-1.55) | | 0.92 | | 1086.5 | | 1105.4 | |  |
|  | Log-additive | | --- | | --- | | --- | | 1.22 (0.98-1.51) | | 0.071 | | 1083.3 | | 1102.2 | |  |
| rs10759243 | |  | | C/C | | 190(34.7%) | | 69 (24%) | | 1.00 | |  | |  | |  | |
|  | | Codominant | | C/A | | 257(46.9%) | | 152 (53%) | | **1.67(1.19-2.36)** | | 0.0028* | | 1063.6 | | 1087.2 | |
|  | |  | | A/A | | 101(18.4%) | | 66 (23%) | | **1.88(1.24-2.86)** | |  | |  | |  | |
|  | | Dominant | | C/C | | 190(34.7%) | | 69 (24%) | | 1.00 | |  | |  | |  | |
|  | |  | | C/A-A/A | | 358(65.3%) | | 218 (76%) | | **1.73(1.25-2.40)** | | 8e-04* | | 1062 | | 1080.9 | |
|  | | Recessive | | C/C-C/A | | 447(81.6%) | | 221 (77%) | | 1.00 | |  | |  | |  | |
|  | |  | | A/A | | 101(18.4%) | | 66 (23%) | | 1.36 (0.95-1.93) | | 0.092 | | 1070.5 | | 1089.4 | |
|  | | Log-additive | | --- | | --- | | --- | | **1.39(1.13-1.70)** | | 0.0016* | | 1063.4 | | 1082.3 | |

| rs704010 |  | G/G | 273(49.7%) | 129(44.2%) | 1.00 |  |  |  |
| --- | --- | --- | --- | --- | --- | --- | --- | --- |
|  | Codominant | G/A | 240(43.7%) | 127(43.5%) | 1.13 (0.84-1.53) | 0.02* | 1080.7 | 1104.4 |
|  |  | A/A | 36 (6.6%) | 36 (12.3%) | **2.08(1.25-3.45)** |  |  |  |
|  | Dominant | G/G | 273(49.7%) | 129(44.2%) | 1.00 |  |  |  |
|  |  | G/A-A/A | 276(50.3%) | 163(55.8%) | 1.26 (0.95-1.68) | 0.11 | 1084 | 1103 |
|  | Recessive | G/G-G/A | 513(93.4%) | 256(87.7%) | 1.00 |  |  |  |
|  |  | A/A | 36 (6.6%) | 36 (12.3%) | **1.95(1.20-3.18)** | 0.0075* | 1079.4 | 1098.3 |
|  | Log-additive | --- | --- | --- | **1.31(1.05-1.64)** | 0.016* | 1080.7 | 1099.6 |

AIC: Akaike’s Information criterion; BIC: Bayesian Information criterion;

**p* value ≤ 0.05 indicates statistical significance

**Table S6** Logistic regression analysis of the association between the SNPs and ER-breast cancer risk (adjusted by Age + BMI).

| **SNP** | | **Model** | | **Genotype** | | **Control** | | **Case** | | **OR(95%CI)** | | | | **P-value** | | **AIC** | **BIC** |
| --- | --- | --- | --- | --- | --- | --- | --- | --- | --- | --- | --- | --- | --- | --- | --- | --- | --- |
| rs4973768 | |  | | C/C | | 329(59.9%) | | 66 (48.5%) | | 1.00 | | | |  | |  |  |
|  | | Codominant | | C/T | | 194(35.3%) | | 59 (43.4%) | | **1.49(1.01-2.22)** | | | | 0.055 | | 684.6 | 707.2 |
|  | |  | | T/T | | 26 (4.7%) | | 11 (8.1%) | | 2.01 (0.94-4.30) | | | |  | |  |  |
|  | | Dominant | | C/C | | 329(59.9%) | | 66 (48.5%) | | 1.00 | | | |  | |  |  |
|  | |  | | C/T-T/T | | 220(40.1%) | | 70 (51.5%) | | **1.56(1.07-2.27)** | | | | 0.022* | | 683.1 | 701.2 |
|  | | Recessive | | C/C-C/T | | 523(95.3%) | | 125(91.9%) | | 1.00 | | | |  | |  |  |
|  | |  | | T/T | | 26 (4.7%) | | 11 (8.1%) | | 1.70 (0.81-3.54) | | | | 0.17 | | 686.5 | 704.6 |
|  | | Log-additive | | --- | | --- | | --- | | **1.45(1.07-1.97)** | | | | 0.016* | | 682.6 | 700.7 |
| rs1432679 | |  | | C/C | | 222(40.4%) | | 66 (48.5%) | | 1.00 | | | |  | |  |  |
|  | | Codominant | | T/C | | 256(46.6%) | | 59 (43.4%) | | 0.78 (0.52-1.16) | | | | 0.12 | | 686.2 | 708.8 |
|  | |  | | T/T | | 71 (12.9%) | | 11 (8.1%) | | 0.52 (0.26-1.04) | | | |  | |  |  |
|  | | Dominant | | C/C | | 222(40.4%) | | 66 (48.5%) | | 1.00 | | | |  | |  |  |
|  | |  | | T/C-T/T | | 327(59.6%) | | 70 (51.5%) | | 0.72 (0.49-1.05) | | | | 0.092 | | 685.5 | 703.6 |
|  | | Recessive | | C/C-T/C | | 478(87.1%) | | 125(91.9%) | | 1.00 | | | |  | |  |  |
|  | |  | | T/T | | 71 (12.9%) | | 11 (8.1%) | | 0.59 (0.30-1.15) | | | | 0.1 | | 685.7 | 703.8 |
|  | | Log-additive | | --- | | --- | | --- | | **0.74(0.56-0.99)** | | | | 0.043* | | 684.3 | 702.4 |
| rs10822013 |  | | C/C | | 171(31.1%) | | 32 (23.5%) | | 1.00 | |  |  |  | |  | | |
|  | Codominant | | C/T | | 268(48.8%) | | 68 (50%) | | 1.35 (0.85-2.14) | | 0.11 | 685.9 | 708.6 | |  | | |
|  |  | | T/T | | 110 (20%) | | 36 (26.5%) | | **1.77(1.04-3.03)** | |  |  |  | |  | | |
|  | Dominant | | C/C | | 171(31.1%) | | 32 (23.5%) | | 1.00 | |  |  |  | |  | | |
|  |  | | C/T-T/T | | 378(68.8%) | | 104(76.5%) | | 1.47 (0.95-2.28) | | 0.076 | 685.2 | 703.3 | |  | | |
|  | Recessive | | C/C-C/T | | 439 (80%) | | 100(73.5%) | | 1.00 | |  |  |  | |  | | |
|  |  | | T/T | | 110 (20%) | | 36 (26.5%) | | 1.46 (0.95-2.26) | | 0.093 | 685.5 | 703.7 | |  | | |
|  | Log-additive | | --- | | --- | | --- | | **1.33(1.02-1.74)** | | 0.035* | 683.9 | 702 | |  | | |

AIC: Akaike’s Information criterion; BIC: Bayesian Information criterion;

**p* value ≤ 0.05 indicates statistical significance

**Table S7 Logistic regression analysis of the association between the SNPs and PR+ breast cancer risk (adjusted by Age + BMI).**

| **SNP** | | **Model** | | **Genotype** | | **Control** | | **Case** | | **OR(95%CI)** | | **P-value** | | **AIC** | | **BIC** | |
| --- | --- | --- | --- | --- | --- | --- | --- | --- | --- | --- | --- | --- | --- | --- | --- | --- | --- |
| rs981782 | |  | | T/T | | 263(47.9%) | | 96 (38.9%) | | 1.00 | |  | |  | |  | |
|  | | Codominant | | G/T | | 226(41.2%) | | 124(50.2%) | | **1.49(1.08-2.05)** | | 0.052 | | 982.1 | | 1005.5 | |
|  | |  | | G/G | | 60 (10.9%) | | 27 (10.9%) | | 1.24 (0.74-2.07) | |  | |  | |  | |
|  | | Dominant | | T/T | | 263(47.9%) | | 96 (38.9%) | | 1.00 | |  | |  | |  | |
|  | |  | | G/T-G/G | | 286(52.1%) | | 151(61.1%) | | **1.44(1.06-1.95)** | | 0.02* | | 980.6 | | 999.3 | |
|  | | Recessive | | T/T-G/T | | 489(89.1%) | | 220(89.1%) | | 1.00 | |  | |  | |  | |
|  | |  | | G/G | | 60 (10.9%) | | 27 (10.9%) | | 1.01 (0.62-1.64) | | 0.97 | | 986 | | 1004.7 | |
|  | | Log-additive | | --- | | --- | | --- | | 1.22 (0.98-1.53) | | 0.081 | | 982.9 | | 1001.7 | |
| rs10759243 | |  | | C/C | | 190(34.7%) | | 61 (25.2%) | | 1.00 | |  | |  | |  | |
|  | | Codominant | | C/A | | 257(46.9%) | | 130(53.7%) | | **1.63(1.13-2.33)** | | 0.017* | | 965.9 | | 989.3 | |
|  | |  | | A/A | | 101(18.4%) | | 51 (21.1%) | | **1.65(1.05-2.58)** | |  | |  | |  | |
|  | | Dominant | | C/C | | 190(34.7%) | | 61 (25.2%) | | 1.00 | |  | |  | |  | |
|  | |  | | C/A-A/A | | 358(65.3%) | | 181(74.8%) | | **1.63 (116-2.30)** | | 0.0043* | | 964 | | 982.6 | |
|  | | Recessive | | C/C-C/A | | 447(81.6%) | | 191(78.9%) | | 1.00 | |  | |  | |  | |
|  | |  | | A/A | | 101(18.4%) | | 51 (21.1%) | | 1.21 (0.83-1.77) | | 0.32 | | 971.1 | | 989.8 | |
|  | | Log-additive | | --- | | --- | | --- | | **1.31(1.05-1.63)** | | 0.015* | | 966.2 | | 984.9 | |
| rs704010 |  | | G/G | | 273(49.7%) | | 104(42.1%) | | 1.00 | |  | |  | |  | |  |
|  | Codominant | | G/A | | 240(43.7%) | | 119(48.2%) | | 1.33 (0.97-1.82) | | 0.078 | | 982.9 | | 1006.3 | |  |
|  |  | | A/A | | 36 (6.6%) | | 24 (9.7%) | | 1.72 (0.97-3.02) | |  | |  | |  | |  |
|  | Dominant | | G/G | | 27(49.7%) | | 104(42.1%) | | 1.00 | |  | |  | |  | |  |
|  |  | | G/A-A/A | | 276(50.3%) | | 143(57.9%) | | **1.38 (1.02-1.87)** | | 0.037* | | 981.6 | | 1000.4 | |  |
|  | Recessive | | G/G-G/A | | 513(93.4%) | | 223(90.3%) | | 1.00 | |  | |  | |  | |  |
|  |  | | A/A | | 36 (6.6%) | | 24 (9.7%) | | 1.49 (0.87-2.56) | | 0.16 | | 984 | | 1002.7 | |  |
|  | Log-additive | | --- | | --- | | --- | | **1.32(1.04-1.67)** | | 0.024* | | 980.9 | | 999.6 | |  |

AIC: Akaike’s Information criterion; BIC: Bayesian Information criterion;

**p* value ≤ 0.05 indicates statistical significance

**Table S8** Logistic regression analysis of the association between the SNPs and PR- breast cancer risk (adjusted by Age + BMI).

| **SNP** | | **Model** | | | **Genotype** | | | **Control** | | | **Case** | | | **OR(95%CI)** | | | **P-value** | | | | **AIC** | | | **BIC** | | |
| --- | --- | --- | --- | --- | --- | --- | --- | --- | --- | --- | --- | --- | --- | --- | --- | --- | --- | --- | --- | --- | --- | --- | --- | --- | --- | --- |
| rs1432679 | |  | | | C/C | | | 222(40.4%) | | | 90 (50%) | | | 1.00 | | |  | | | |  | | |  | | |
|  | | Codominant | | | T/C | | | 256(46.6%) | | | 75 (41.7%) | | | 0.72 (0.51-1.03) | | | 0.047* | | | | 816.5 | | | 839.4 | | |
|  | |  | | | T/T | | | 71 (12.9%) | | | 15 (8.3%) | | | **0.52(0.28-0.96)** | | |  | | | |  | | |  | | |
|  | | Dominant | | | C/C | | | 222(40.4%) | | | 90 (50%) | | | 1.00 | | |  | | | |  | | |  | | |
|  | |  | | | T/C-T/T | | | 327(59.6%) | | | 90 (50%) | | | **0.68(0.48-0.95)** | | | 0.026* | | | | 815.6 | | | 834 | | |
|  | | Recessive | | | C/C-T/C | | | 478(87.1%) | | | 165(91.7%) | | | 1.00 | | |  | | | |  | | |  | | |
|  | |  | | | T/T | | | 71 (12.9%) | | | 15 (8.3%) | | | 0.61 (0.34-1.10) | | | 0.089 | | | | 817.7 | | | 836 | | |
|  | | Log-additive | | | --- | | | --- | | | --- | | | **0.72(0.56-0.94)** | | | 0.014* | | | | 814.5 | | | 832.8 | | |
| rs10759243 | | |  | | | C/C | | | 190(34.7%) | | | 47 (26.7%) | | | 1.00 | | |  | |  | | |  | | |  |
|  | | | Codominant | | | C/A | | | 257(46.9%) | | | 85 (48.3%) | | | 1.32 (0.88-1.97) | | | 0.069 | | 806 | | | 829 | | |  |
|  | | |  | | | A/A | | | 101(18.4%) | | | 44 (25%) | | | **1.76(1.09-2.84)** | | |  | |  | | |  | | |  |
|  | | | Dominant | | | C/C | | | 190(34.7%) | | | 47 (26.7%) | | | 1.00 | | |  | |  | | |  | | |  |
|  | | |  | | | C/A-A/A | | | 358(65.3%) | | | 129(73.3%) | | | 1.44 (0.99-2.10) | | | 0.056 | | 805.7 | | | 824.1 | | |  |
|  | | | Recessive | | | C/C-C/A | | | 447(81.6%) | | | 132 (75%) | | | 1.00 | | |  | |  | | |  | | |  |
|  | | |  | | | A/A | | | 101(18.4%) | | | 44 (25%) | | | 1.49 (0.99-2.23) | | | 0.059 | | 805.8 | | | 824.2 | | |  |
|  | | | Log-additive | | | --- | | | --- | | | --- | | | **1.33(1.04-1.68)** | | | 0.021* | | 804 | | | 822.4 | | |  |
| rs704010 |  | | | G/G | | | 273(49.7%) | | | 90 (50%) | | | 1.00 | | |  | | |  | | |  | | |  | |
|  | Codominant | | | G/A | | | 240(43.7%) | | | 67 (37.2%) | | | 0.85 (0.59-1.22) | | | 0.027* | | | 815.4 | | | 838.3 | | |  | |
|  |  | | | A/A | | | 36 (6.6%) | | | 23 (12.8%) | | | **1.94(1.09-3.45)** | | |  | | |  | | |  | | |  | |
|  | Dominant | | | G/G | | | 273(49.7%) | | | 90 (50%) | | | 1.00 | | |  | | |  | | |  | | |  | |
|  |  | | | G/A-A/A | | | 276(50.3%) | | | 90 (50%) | | | 0.99 (0.71-1.39) | | | 0.96 | | | 820.6 | | | 838.9 | | |  | |
|  | Recessive | | | G/G-G/A | | | 513(93.4%) | | | 157(87.2%) | | | 1.00 | | |  | | |  | | |  | | |  | |
|  |  | | | A/A | | | 36 (6.6%) | | | 23 (12.8%) | | | **2.09(1.20-3.63)** | | | 0.012* | | | 814.2 | | | 832.5 | | |  | |
|  | Log-additive | | | --- | | | --- | | | --- | | | 1.16 (0.89-1.50) | | | 0.28 | | | 819.4 | | | 837.8 | | |  | |

AIC: Akaike’s Information criterion; BIC: Bayesian Information criterion;

**p* value ≤ 0.05 indicates statistical significance

**Table S9** Logistic regression analysis of the association between the SNPs and Clinic stage (UICC) I and II breast cancer risk (adjusted by Age + BMI).

| **SNP** | **Model** | **Genotype** | **Control** | **Case** | **OR(95%CI)** | **P-value** | **AIC** | **BIC** |
| --- | --- | --- | --- | --- | --- | --- | --- | --- |
| rs10759243 |  | C/C | 190(34.7%) | 80 (27.1%) | 1.00 |  |  |  |
|  | Codominant | C/A | 257(46.9%) | 143(48.5%) | 1.33 (0.95-1.86) | 0.023* | 1090.2 | 1113.8 |
|  |  | A/A | 101(18.4%) | 72 (24.4%) | **1.74(1.17-2.61)** |  |  |  |
|  | Dominant | C/C | 190(34.7%) | 80 (27.1%) | 1.00 |  |  |  |
|  |  | C/A-A/A | 358(65.3%) | 215(72.9%) | **1.45(1.06-1.98)** | 0.02* | 1090.2 | 1109.2 |
|  | Recessive | C/C-C/A | 447(81.6%) | 223(75.6%) | 1.00 |  |  |  |
|  |  | A/A | 101(18.4%) | 72 (24.4%) | **1.46(1.04-2.07)** | 0.031* | 1091 | 1110 |
|  | Log-additive | --- | --- | --- | **1.32(1.08-1.61)** | 0.0061* | 1088.2 | 1107.1 |

| rs704010 |  | G/G | 273(49.7%) | 140(46.4%) | 1.00 |  |  |  |
| --- | --- | --- | --- | --- | --- | --- | --- | --- |
|  | Codominant | G/A | 240(43.7%) | 126(41.7%) | 1.03 (0.76-1.39) | 0.042* | 1107.1 | 1130.8 |
|  |  | A/A | 36 (6.6%) | 36 (11.9%) | **1.90(1.15-3.15)** |  |  |  |
|  | Dominant | G/G | 273(49.7%) | 140(46.4%) | 1.00 |  |  |  |
|  |  | G/A-A/A | 276(50.3%) | 162(53.6%) | 1.15 (0.86-1.52) | 0.34 | 1110.5 | 1129.5 |
|  | Recessive | G/G-G/A | 513(93.4%) | 266(88.1%) | 1.00 |  |  |  |
|  |  | A/A | 36 (6.6%) | 36 (11.9%) | **1.88(1.15-3.05)** | 0.012* | 1105.1 | 1124.1 |
|  | Log-additive | --- | --- | --- | 1.23 (0.99-1.53) | 0.065 | 1108 | 1127 |

AIC: Akaike’s Information criterion; BIC: Bayesian Information criterion;

**p* value ≤ 0.05 indicates statistical significance

**Table S10** Logistic regression analysis of the association between the SNPs and Clinic stage (UICC) III and IV breast cancer risk (adjusted by Age + BMI).

| **SNP** | **Model** | | **Genotype** | | | | **Control** | | | **Case** | | | **OR(95%CI)** | | | **P-value** | | | **AIC** | | | **BIC** | | |
| --- | --- | --- | --- | --- | --- | --- | --- | --- | --- | --- | --- | --- | --- | --- | --- | --- | --- | --- | --- | --- | --- | --- | --- | --- |
| rs4849887 |  | | C/C | | | | 367(66.8%) | | | 78 (65.5%) | | | 1.00 | | |  | | |  | | |  | | |
|  | Codominant | | T/C | | | | 164(29.9%) | | | 32 (26.9%) | | | 0.93 (0.59-1.46) | | | 0.14 | | | 631.4 | | | 653.9 | | |
|  |  | | T/T | | | | 18 (3.3%) | | | 9 (7.6%) | | | **2.33(1.01-5.39)** | | |  | | |  | | |  | | |
|  | Dominant | | C/C | | | | 367(66.8%) | | | 78 (65.5%) | | | 1.00 | | |  | | |  | | |  | | |
|  |  | | T/C-T/T | | | | 182(33.1%) | | | 41 (34.5%) | | | 1.07 (0.70-1.62) | | | 0.76 | | | 633.3 | | | 651.3 | | |
|  | Recessive | | C/C-T/C | | | | 531(96.7%) | | | 110(92.4%) | | | 1.00 | | |  | | |  | | |  | | |
|  |  | | T/T | | | | 18 (3.3%) | | | 9 (7.6%) | | | **2.39(1.04-5.46)** | | | 0.05* | | | 629.5 | | | 647.5 | | |
|  | Log-additive | | --- | | | | --- | | | --- | | | 1.19 (0.85-1.68) | | | 0.32 | | | 632.4 | | | 650.4 | | |
| rs981782 |  | | T/T | | 263(47.9%) | | | 43 (36.1%) | | | 1.00 | | |  | | | |  | | |  | | |  |
|  | Codominant | | G/T | | 226(41.2%) | | | 68 (57.1%) | | | **1.83(1.20-2.80)** | | | 0.0063* | | | | 625.2 | | | 647.8 | | |  |
|  |  | | G/G | | 60 (10.9%) | | | 8 (6.7%) | | | 0.82 (0.37-1.84) | | |  | | | |  | | |  | | |  |
|  | Dominant | | T/T | | 263(47.9%) | | | 43 (36.1%) | | | 1.00 | | |  | | | |  | | |  | | |  |
|  |  | | G/T-G/G | | 286(52.1%) | | | 76 (63.9%) | | | **1.62(1.08-2.45)** | | | 0.019* | | | | 627.9 | | | 645.9 | | |  |
|  | Recessive | | T/T-G/T | | 489(89.1%) | | | 111(93.3%) | | | 1.00 | | |  | | | |  | | |  | | |  |
|  |  | | G/G | | 60 (10.9%) | | | 8 (6.7%) | | | 0.59 (0.28-1.28) | | | 0.16 | | | | 631.4 | | | 649.4 | | |  |
|  | Log-additive | | --- | | --- | | | --- | | | 1.19 (0.88-1.60) | | | 0.25 | | | | 632.1 | | | 650.1 | | |  |
| rs10759243 | |  | | C/C | | 190(34.7%) | | | 27 (23.1%) | | | 1.00 | | |  | |  | | |  | | |  | |
|  | | Codominant | | C/A | | 257(46.9%) | | | 67 (57.3%) | | | **1.85(1.14-3.00)** | | | 0.037* | | 621.4 | | | 643.9 | | |  | |
|  | |  | | A/A | | 101(18.4%) | | | 23 (19.7%) | | | 1.63 (0.89-3.00) | | |  | |  | | |  | | |  | |
|  | | Dominant | | C/C | | 190(34.7%) | | | 27 (23.1%) | | | 1.00 | | |  | |  | | |  | | |  | |
|  | |  | | C/A-A/A | | 358(65.3%) | | | 90 (76.9%) | | | **1.79(1.12-2.85)** | | | 0.012* | | 619.6 | | | 637.6 | | |  | |
|  | | Recessive | | C/C-C/A | | 447(81.6%) | | | 94 (80.3%) | | | 1.00 | | |  | |  | | |  | | |  | |
|  | |  | | A/A | | 101(18.4%) | | | 23 (19.7%) | | | 1.10 (0.66-1.83) | | | 0.71 | | 625.8 | | | 643.8 | | |  | |
|  | | Log-additive | | --- | | --- | | | --- | | | 1.31 (0.98-1.74) | | | 0.064 | | 622.5 | | | 640.5 | | |  | |

AIC: Akaike’s Information criterion; BIC: Bayesian Information criterion;

**p* value ≤ 0.05 indicates statistical significance
